# Supplementary figures and images for: Novel kinase regulators of extracellular matrix internalisation identified by high-content screening modulate invasive carcinoma cell migration
Source: PLoS Biol. 2024 Dec 12;22(12):e3002930. doi: 10.1371/journal.pbio.3002930 (PMC11637276; doi:10.1371/journal.pbio.3002930)

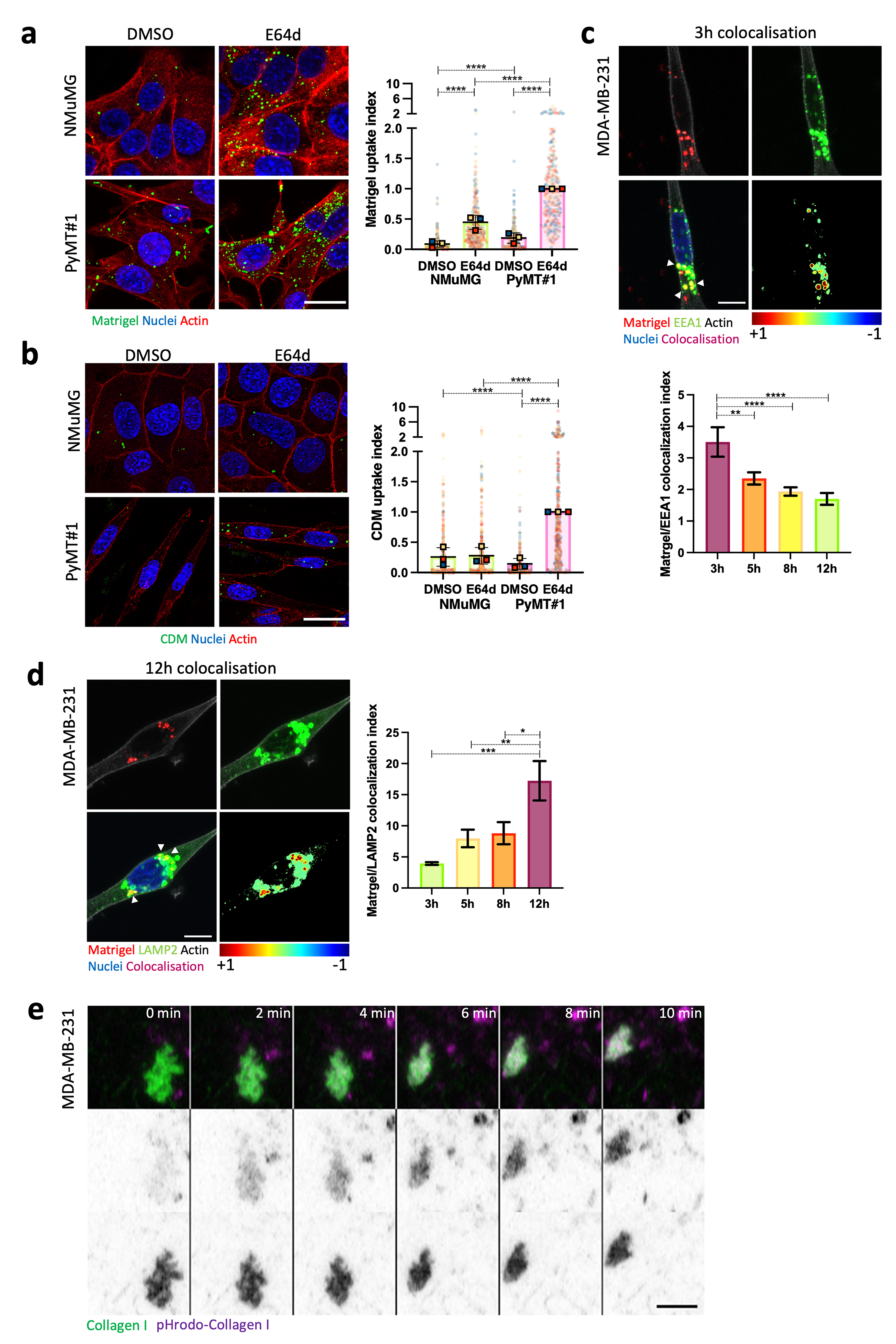

Supplement: S1 Fig — (a) NMuMG and PyMT#1 cells were seeded on NHS Fluorescein-labelled 1 mg/ml matrigel for 12 h in the presence or absence of 20 μM E64d, fixed, stained for actin and nuclei and imaged with a Nikon A1 confocal microscope. Scale bar, 20 μM. Matrigel uptake index was calculated with Image J. Data are presented as the normalised mean ± SD; N = 3 independent experiments. ****p < 0.0001; Kruskal–Wallis test. (b) NMuMG and PyMT#1 cells were seeded on biotinylated CDM for 12 h in the presence or absence of 20 μm E64d, fixed, stained with streptavidin Alexa Fluor 488, Phalloidin Alexa Fluor 555 and DAPI, imaged and quantified as in (a). Scale bar, 20 μm. Data are presented as the normalised mean ± SD; N = 3 independent experiments. ****p < 0.0001; Kruskal–Wallis test. (c, d) MDA-MB-231 cells were seeded on NHS Alexa Fluor 555-labelled 1 mg/ml matrigel for 3 h, 5 h, 8 h, and 12 h, fixed, stained for EEA1 (c) or LAMP2 (d), actin and nuclei and imaged as in (a). Scale bar, 10 μm. Colocalisation was quantified with Image J. Data are presented as the mean ± SEM; N = 3 independent experiments. **p = 0.0057, ****p < 0.0001 (c); *p = 0.0207, **p = 0.0069, ***p = 0.0003 (d); One-way ANOVA/Tukey’s multiple comparisons test. (e) MDA-MB-231 cells were seeded on NHS-fluorescein (green) and pHrodo-labelled (magenta) 1 mg/ml collagen I and imaged live for 5 h. Representative time frames from S1 Video are shown. Scale bar, 5 μm. All the raw data associated with this figure are available in S8 Data. (TIF) [file pbio.3002930.s001.tif]

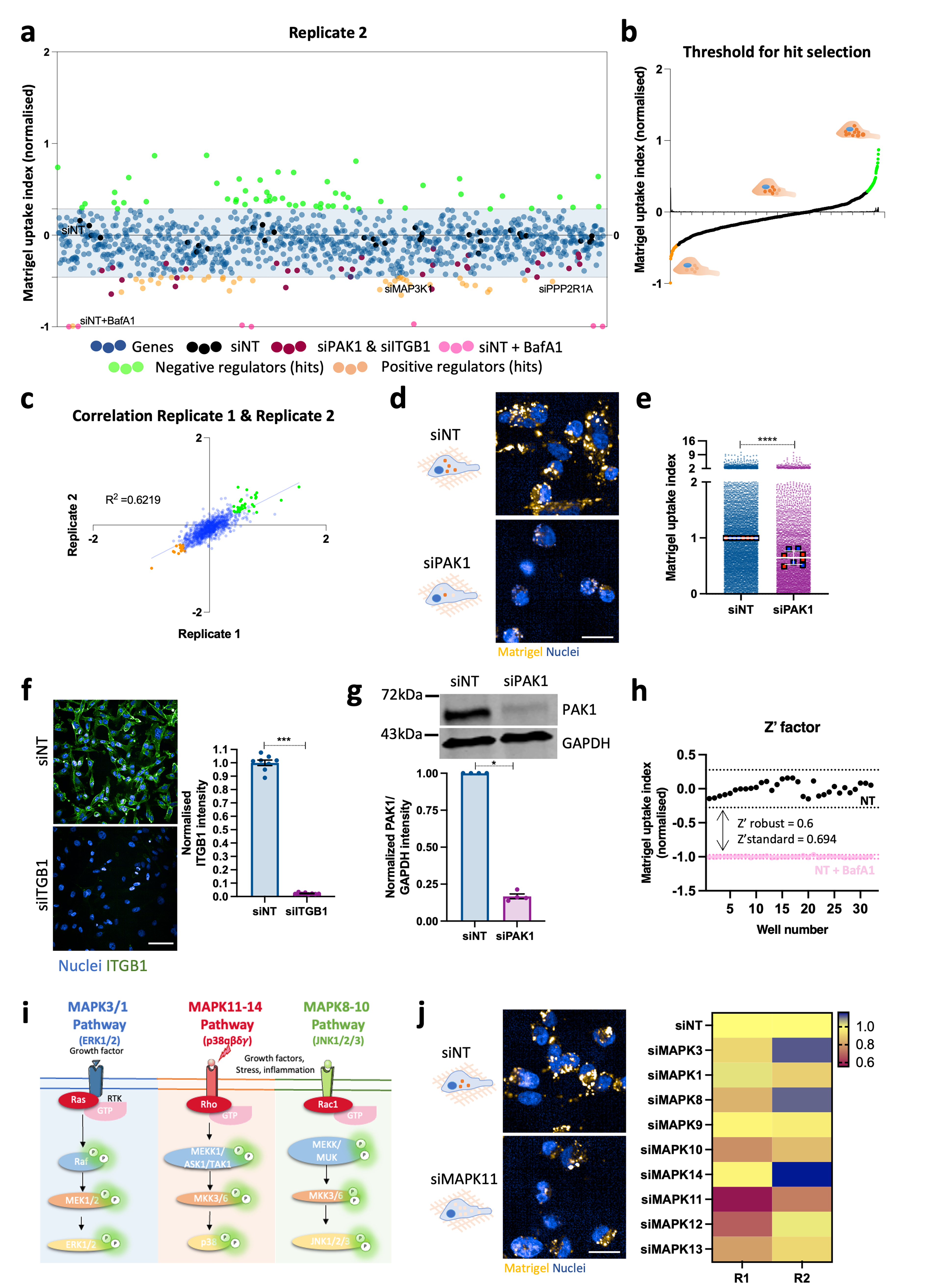

Supplement: S2 Fig — (a) Normalised cloud plot analysis from replicate 2. (b) First derivative of the curve for hit threshold for replicate 2. (c) Correlation between replicate 1 and 2. (d) Representative images of matrigel uptake in cells transfected with a non-targeting siRNA control (siNT) and an siRNA targeting PAK1 (siPAK1). Scale bar, 20 μm. (e) Data are presented as the normalised mean ± SD; N = 6 replicates from 2 independent experiments. ****p < 0.0001; Kruskal–Wallis test. (f) Screening plates were fixed and stained for β1 integrin (green) and nuclei (blue) and imaged with 40× Opera Phenix microscope; scale bar, 60 μm. Signal intensity was quantified with Columbus Software. *p = 0.0286; Mann–Whitney test. (g) MDA-MB-231 cells were transfected with an siRNA targeting PAK1 (siPAK1) or a non-targeting siRNA control (siNT). PAK1 and GAPDH protein levels was quantified by western blotting. Data are presented as the normalised mean ± SD; N = 4 independent experiments; *p = 0.0286; Mann–Whitney test. (h) Z’ robust and standard for screening validation. (i) Diagram of MAPK activation pathways. (j) Heatmap of major MAPKs in the kinome and phosphatome screen. N = 2 biological replicates. Representative images for MAPK11. Scale bar, 20 μm. All the raw data associated with this figure are available in S9 Data. (TIF) [file pbio.3002930.s002.tif]

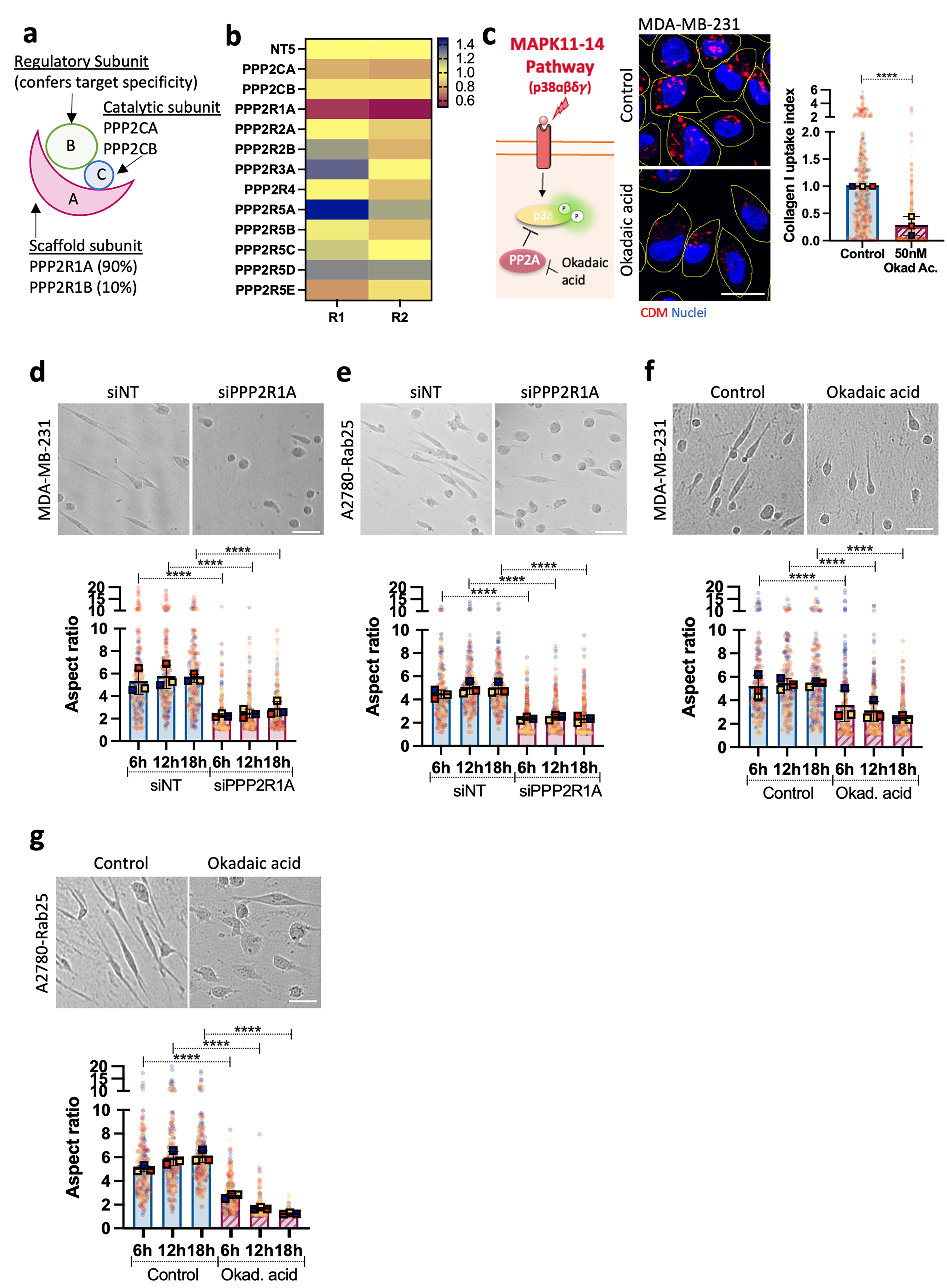

Supplement: S3 Fig — (a) Representative scheme of Protein Phosphatase 2A (PP2A) subunits. (b) Heatmap of major PP2A subunits in the kinome and phosphatome screen. N = 2 biological replicates. (c) 3 × 10^5^ MDA-MB-231 cells were cultured on pHrodo-labelled collagen I for 6 h in the presence of 50 nM okadaic acid (Okad Ac.) or water (Control), stained with 1 μg/ml Hoechst and imaged live. Scale bar, 20 μm. Collagen I uptake index was measured with Image J. Values represented are normalised mean + SD from N = 3 independent experiments; ****p < 0.0001; Kruskal–Wallis test. (d–g) MDA-MB-231 (d) and A2780-Rab25 (e) cells were transfected with an siRNA targeting PPP2R1A (siPPP2R1A) or a non-targeting siRNA control (siNT) and seeded on CDM. MDA-MB-231 (f) and A2780-Rab25 (g) were seeded on CDM in the presence of 50 nM Okadaic acid or the control (water). Cells were imaged live with a 10× Nikon Inverted Ti eclipse with Oko-lab environmental control chamber for 17 h. Aspect ratio (AR) was calculated with Image J at 6 h, 12 h, and 18 h after cell seeding. Scale bar, 50 μm. Values represented are AR from single cells, mean AR + SD from N = 3 independent experiments; ****p < 0.0001; Kruskal–Wallis test. All the raw data associated with this figure are available in S10 Data. (TIF) [file pbio.3002930.s003.tif]

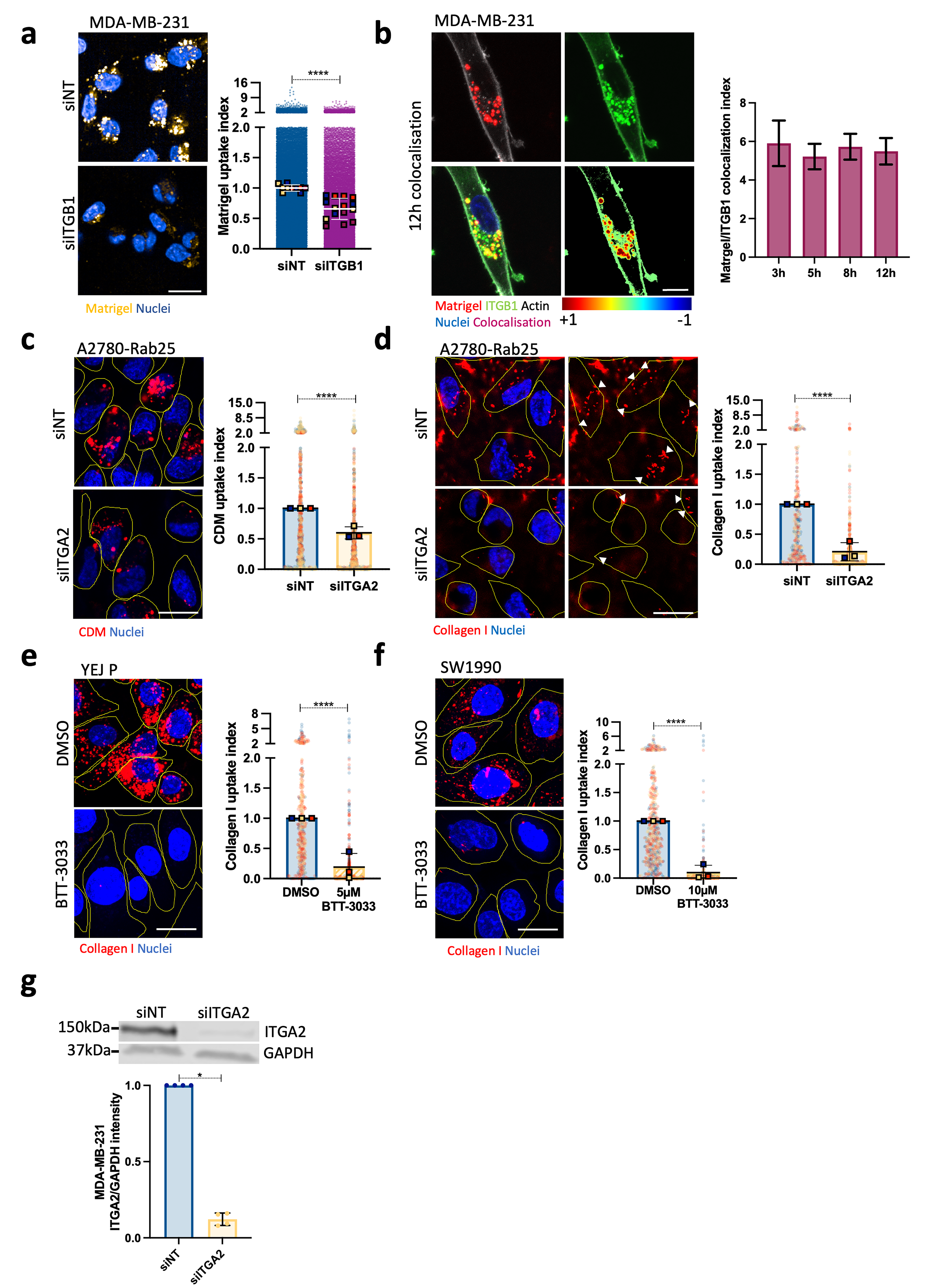

Supplement: S4 Fig — (a) MDA-MB-231 cells transfected with an siRNA targeting β1 integrin (siITGB1) or a non-targeting siRNA control (siNT), plated on pHrodo-labelled 0.5 mg/ml matrigel for 6 h, stained with 1 μg/ml Hoechst and imaged live. Data are presented as the normalised mean ± SD; N = 3 independent experiments. ****p < 0.0001; Mann–Whitney test. (b) MDA-MB-231 cells were seeded on NHS Alexa Fluor 555-labelled 1 mg/ml matrigel for 3 h, 5 h, 8 h, and 12 h, fixed and stained for β1 integrin (ITGB1), actin and nuclei. Scale bar, 10 μm. Data are presented as the mean ± SEM; N = 3 independent experiments. (c, d) A2780-Rab25 cells were transfected with an siRNA targeting α2 integrin (siITGA2) or a non-targeting siRNA control (siNT), seeded on pHrodo-labelled CDM (c) or collagen I (d) for 6 h, stained with 1 μg/ml Hoechst and imaged live. Data are presented as the normalised mean ± SD; N = 3 independent experiments. ****p < 0.0001; Mann–Whitney test. (e) YEJ P cells were allowed to adhere to pHrodo-labelled 1 mg/ml collagen I for 2 h, treated with 5 μM BTT-3033 or DMSO for 14 h, stained with 1 μg/ml Hoechst and imaged live. Data are presented as the normalised mean ± SD; N = 3 independent experiments. ****p < 0.0001; Mann–Whitney test. (f) SW1990 cells were seeded on pHrodo-labelled 1 mg/ml collagen I for 2 h, treated with 10 μM BTT-3033 or DMSO for 4 h, stained with 1 μg/ml Hoechst and imaged live. Data are presented as the normalised mean ± SD; N = 3 independent experiments. ****p < 0.0001; Mann–Whitney test. (g) Cells were transfected with an siRNA targeting α2 integrin (siITGA2) or a non-targeting siRNA control (siNT) for 72 h, lysed and α2 integrin and GAPDH protein levels were measured by western blotting. Data are presented as the normalised mean ± SD; N = 4 independent replicates. *p = 0.0286; Mann–Whitney test. All the raw data associated with this figure are available in S11 Data. (TIF) [file pbio.3002930.s004.tif]

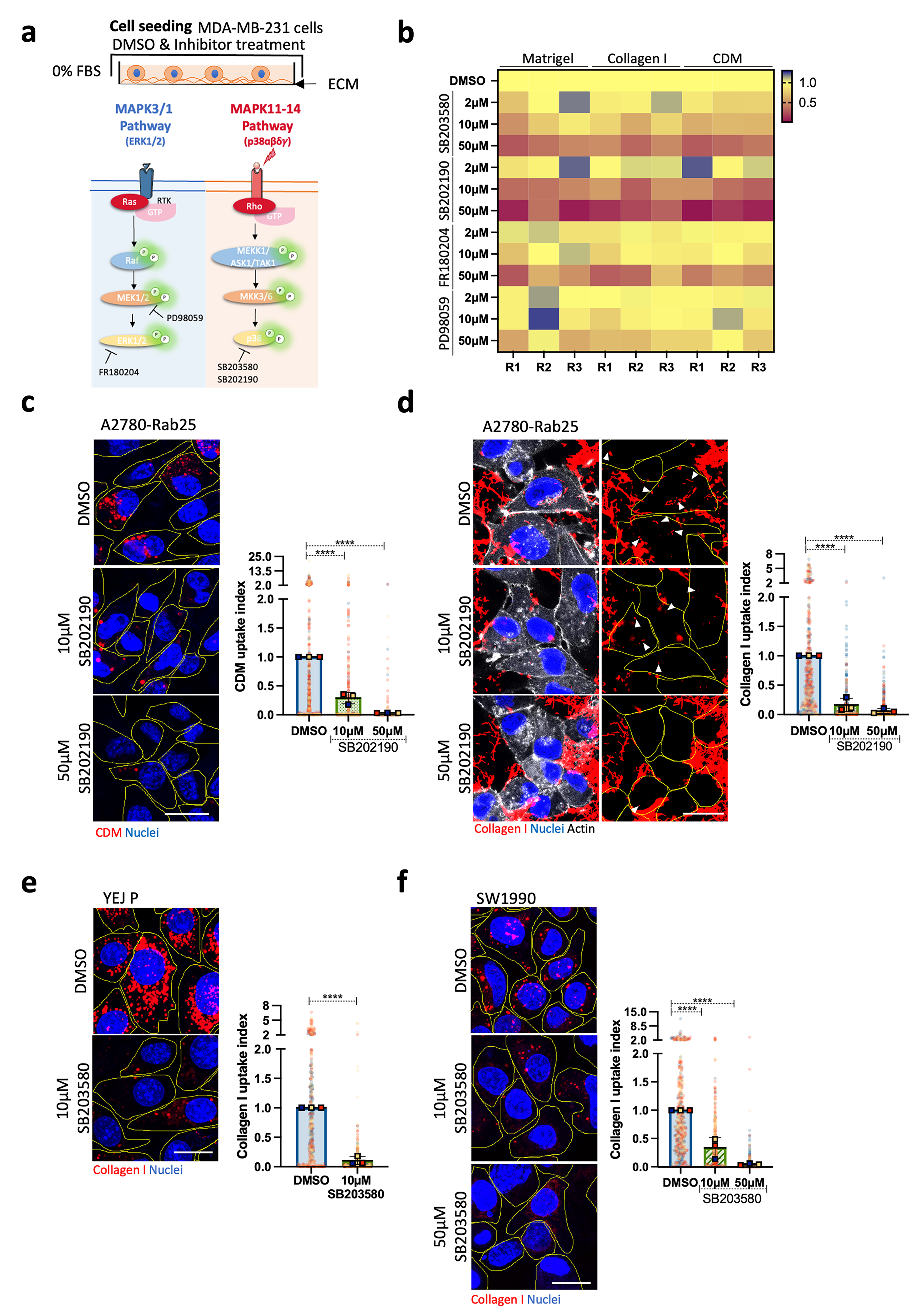

Supplement: S5 Fig — (a) Schematic representation of MAPK inhibitors. (b) MDA-MB-231 cells were serum starved for 16 to 18 h, and 104 cells were seeded on pHrodo-labelled ECM for 6 h in the presence of DMSO or MAPK inhibitors in 0% FBS, stained with 1 μg/ml Hoechst and imaged live. Data analysis was performed with Columbus software. (c) A2780-Rab25 cells were serum starved for 16 to 18 h; 3 × 10^5^ cells were seeded on pHrodo-labelled CDM for 6 h in the presence of DMSO, 10 μM or 50 μM SB202190 in 5% FBS, stained with Hoechst and imaged live. Scale bar, 20 μm. CDM uptake was quantified with ImageJ. Values represented are normalised mean + SD from N = 3 independent experiments; ****p < 0.0001; Kruskal–Wallis test. (d) A2780-Rab25 cells were serum starved for 16 to 18 h, and 3 × 10^5^ cells were cultured on 1 mg/ml collagen I, labelled with NHS-Alexa fluor 555, for 6 h in the presence of DMSO, 10 μM or 50 μM SB202190 in 5% FBS. Cells were fixed and stained for actin and nuclei. Scale bar, 20 μm. Collagen I uptake was quantified with ImageJ. Values represented are normalised mean + SD from N = 3 independent experiments; ****p < 0.0001; Kruskal–Wallis test. (e) YEJ P cells were serum starved for 18 h, and 3 × 10^5^ cells were seeded on pHrodo-labelled 1 mg/ml collagen I for 16 h in the presence of DMSO or 10 μM SB203580 in 5% FBS, stained with Hoechst and imaged live. Scale bar, 20 μm. Collagen I uptake was quantified with ImageJ. Values represented are normalised mean + SD from N = 3 independent experiments; ****p < 0.0001; Mann–Whitney test. (f) SW1990 cells were serum starved for 18 h, and 3 × 10^5^ cells were cultured on pHrodo-labelled 1 mg/ml collagen I for 6 h in the presence of DMSO, 10 μM or 50 μM SB203580 in 5% FBS, stained with Hoechst and imaged live. Scale bar, 20 μm. Collagen I uptake was quantified with ImageJ. Values represented are normalised mean + SD from N = 3 independent experiments; ****p < 0.0001; Kruskal–Wallis test. All the raw data associated with this figure are available [file pbio.3002930.s005.tif]

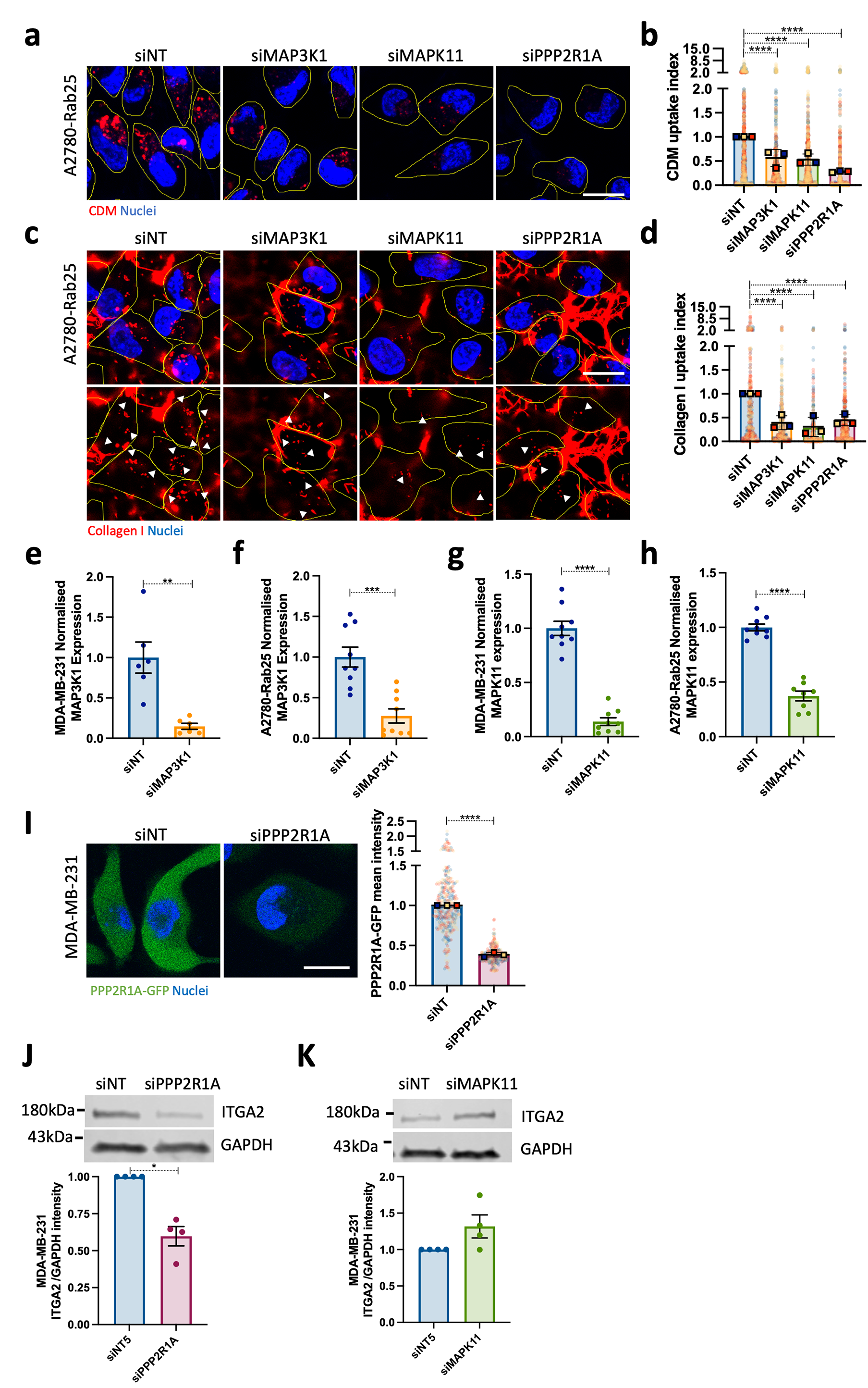

Supplement: S6 Fig — (a) A2780-Rab25 cells were transfected with an siRNA targeting MAPK3K1 (siMAP3K1), an siRNA targeting MAPK11 (siMAPK11), an siRNA targeting PPP2R1A (siPPP2R1A), or a non-targeting siRNA control (siNT), seeded on pHrodo-labelled CDM for 6 h, stained with 1 μg/ml Hoechst and imaged live. Scale bar, 20 μm. (b) CDM uptake index was calculated with Image J. Values represented are normalised mean + SD from N = 3 independent experiments; ****p < 0.0001; Kruskal–Wallis test. (c) A2780-Rab25 cells were transfected as in (a), seeded on 1 mg/ml collagen I, labelled with NHS-Alexa fluor 555, for 6 h, fixed and stained for nuclei. Scale bar, 20 μm. (d) Collagen I uptake index was calculated with Image J. Values represented are normalised mean + SD from N = 3 independent experiments; ****p < 0.0001; Kruskal–Wallis test. (e, f) MDA-MB-231 (e) and A2780-Rab25 (f) cells were transfected with an siRNA targeting MAP3K1 (siMAP3K1) or a non-targeting siRNA control (siNT), RNA was extracted and MAP3K1 expression was quantified by qPCR. Normalised data from N = 3 independent experiments; **p = 0.0022; ***p = 0.0003; Mann–Whitney test. (g, h) MDA-MB-231 (g) and A2780-Rab25 (h) cells were transfected with an siRNA targeting MAPK11 (siMAPK11) or a non-targeting siRNA control (siNT), RNA was extracted and MAPK11 expression was quantified by qPCR. Normalised data from N = 3 independent experiments; ****p < 0.0001; Kruskal–Wallis test. (i) MDA-MB-231 cells overexpressing PPP2R1A-GFP were transfected with an siRNA targeting PPP2R1A (siPPP2R1A) or a non-targeting siRNA control (siNT), fixed and stained for nuclei. GFP normalised mean intensity + SD from N = 3 independent experiments is shown; ****p < 0.0001; Mann–Whitney test. (j, k) MDA-MB-231 cells were transfected with an siRNA targeting MAPK11 (siMAPK11, j), an siRNA targeting PPP2R1A (siPPP2R1A, k) or a non-targeting siRNA control (siNT), α2 integrin (ITGA2) and GAPDH protein levels was quantified by western blotting. Data are presented as t [file pbio.3002930.s006.tif]

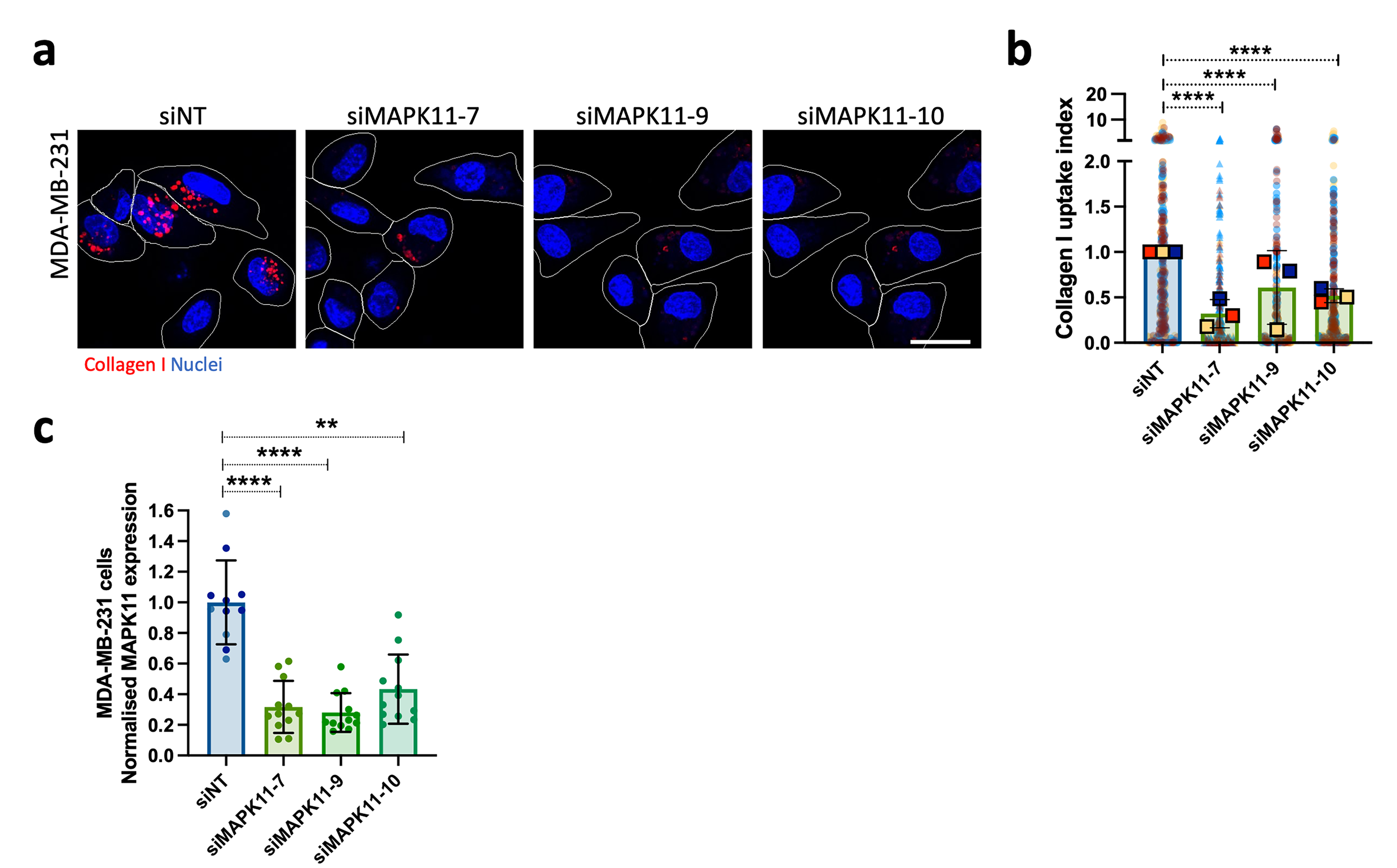

Supplement: S7 Fig — (a) MDA-MB-231 cells were transfected with 3 individual siRNA targeting MAPK11 (siMAPK11-7, 9, and 10) or a non-targeting siRNA control (siNT), seeded on pHrodo-labelled 1 mg/ml collagen I for 6 h, stained with 1 μg/ml Hoechst and imaged live. Scale bar, 20 μm. (b) Collagen I uptake index was calculated with Image J. Values represented are normalised mean + SD from N = 3 independent experiments; ****p < 0.0001; Kruskal–Wallis test. (c) MDA-MB-231 cells were transfected as in (a), RNA was extracted and MAPK11 expression was quantified by qPCR. Normalised data from N = 4 independent experiments; ****p < 0.0001; **p = 0.0065; Kruskal–Wallis test. All the raw data associated with this figure are available in S14 Data. (TIF) [file pbio.3002930.s007.tif]

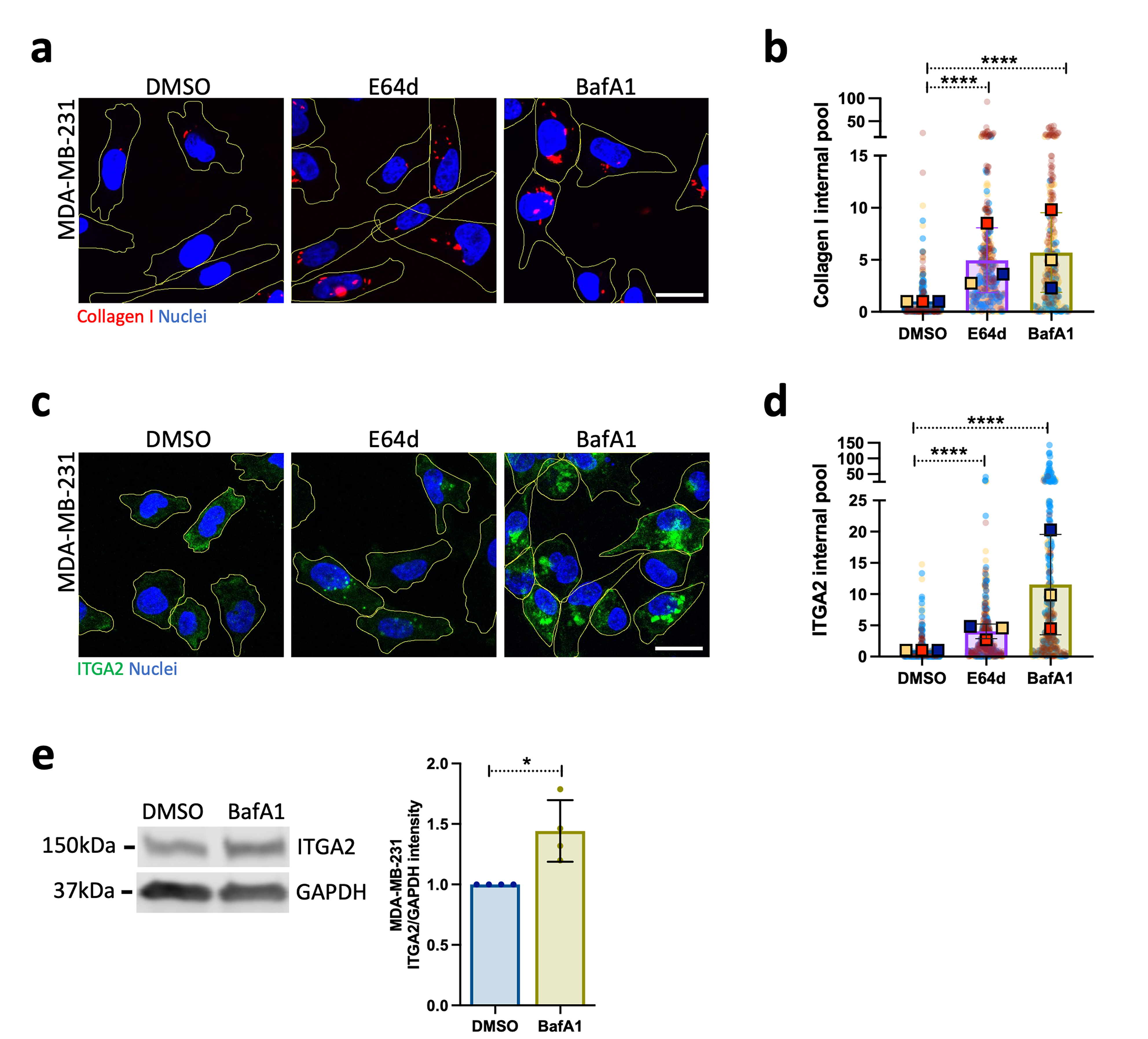

Supplement: S8 Fig — (a) MDA-MB-231 cells were seeded on NHS-Alexa Fluor 555-labelled 1 mg/ml collagen I for 24 h in the presence of 20 mM E64d, 200 nM Bafilomycin A1 (BafA1) or DMSO control, stained with 1 μg/ml Hoechst and imaged live. Scale bar, 20 μm. (b) Collagen I internal pool was calculated with Image J. Values represented are normalised mean + SD from N = 3 independent experiments; ****p < 0.0001; Kruskal–Wallis test. (c) MDA-MB-231 cells treated as in (a), fixed and stained for α2 integrin (ITGA2, green), actin (red), and nuclei (blue). Scale bar, 20 μm. (d) α2 integrin internal pool was calculated with Image J. Values represented are normalised mean + SD from N = 3 independent experiments; ****p < 0.0001; Kruskal–Wallis test. (e) MDA-MB-231 cells were seeded on 1 mg/ml collagen I for 24 h in the presence of 200 nM Bafilomycin A1 (BafA1) or DMSO control, α2 integrin (ITGA2), and GAPDH protein levels was quantified by western blotting. Data are presented as the normalised mean ± SD; N = 4 independent experiments; *p = 0.0286; Mann–Whitney test. All the raw data associated with this figure are available in S15 Data. (TIF) [file pbio.3002930.s008.tif]

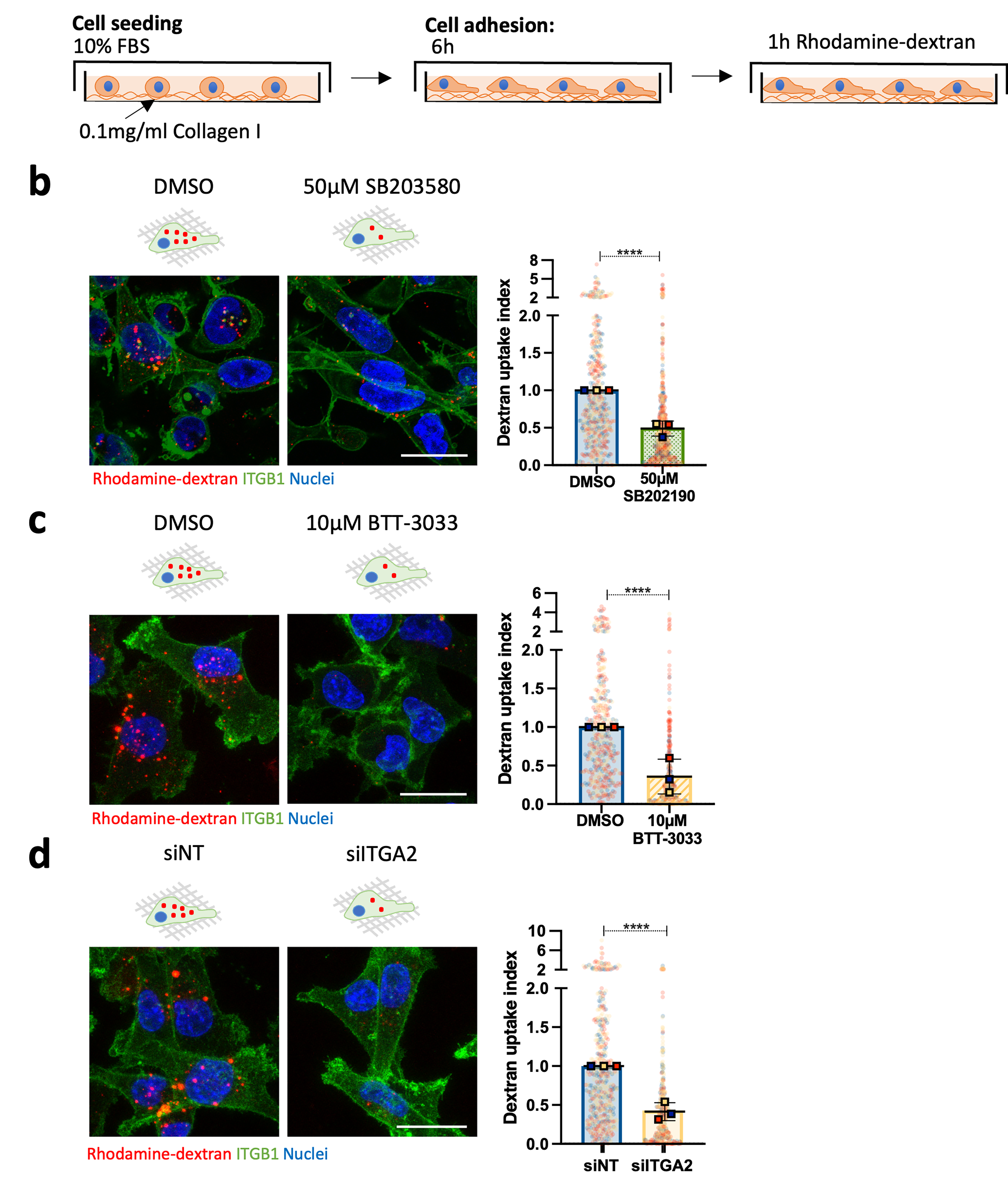

Supplement: S9 Fig — (a) Schematic representation of the experimental set up. (b) MDA-MB-231 cells were seeded on 0.1 mg/ml collagen I for 6 h, pretreated with DMSO (vehicle) or 50 μm SB202190 for 30 min, incubated with 0.25 mg/ml rhodamine-dextran (red) for 1 h in the presence of DMSO or SB202190, fixed and stained for β1 integrin (ITGB1) and nuclei. Scale bar, 20 μM. Dextran uptake index was measured with Image J. Data are presented as the normalised mean ± SD; N = 3 independent experiments. ****p < 0.0001; Mann–Whitney test. (c) MDA-MB-231 cells were seeded on 0.1 mg/ml collagen I for 6 h in the presence of DMSO (vehicle) or 10 μM BTT-3033, incubated with 0.25 mg/ml rhodamine-dextran (red) for 1 h, fixed and stained for β1 integrin (ITGB1) and nuclei. Scale bar, 20 μm. Dextran uptake index was measured with Image J. Data are presented as the normalised mean ± SD; N = 3 independent experiments. ****p < 0.0001; Mann–Whitney test. (d) MDA-MB-231 cells were transfected with an siRNA targeting α2 integrin (siITGA2) or a non-targeting siRNA control (siNT), seeded on 0.1 mg/ml collagen I for 6 h, incubated with 0.25 mg/ml rhodamine-dextran (red) for 1 h, fixed and stained for β1 integrin (ITGB1) and nuclei. Scale bar, 20 μm. Data are presented as the normalised mean ± SD; N = 3 independent experiments. ****p < 0.0001; Mann–Whitney test. All the raw data associated with this figure are available in S16 Data. (TIF) [file pbio.3002930.s009.tif]

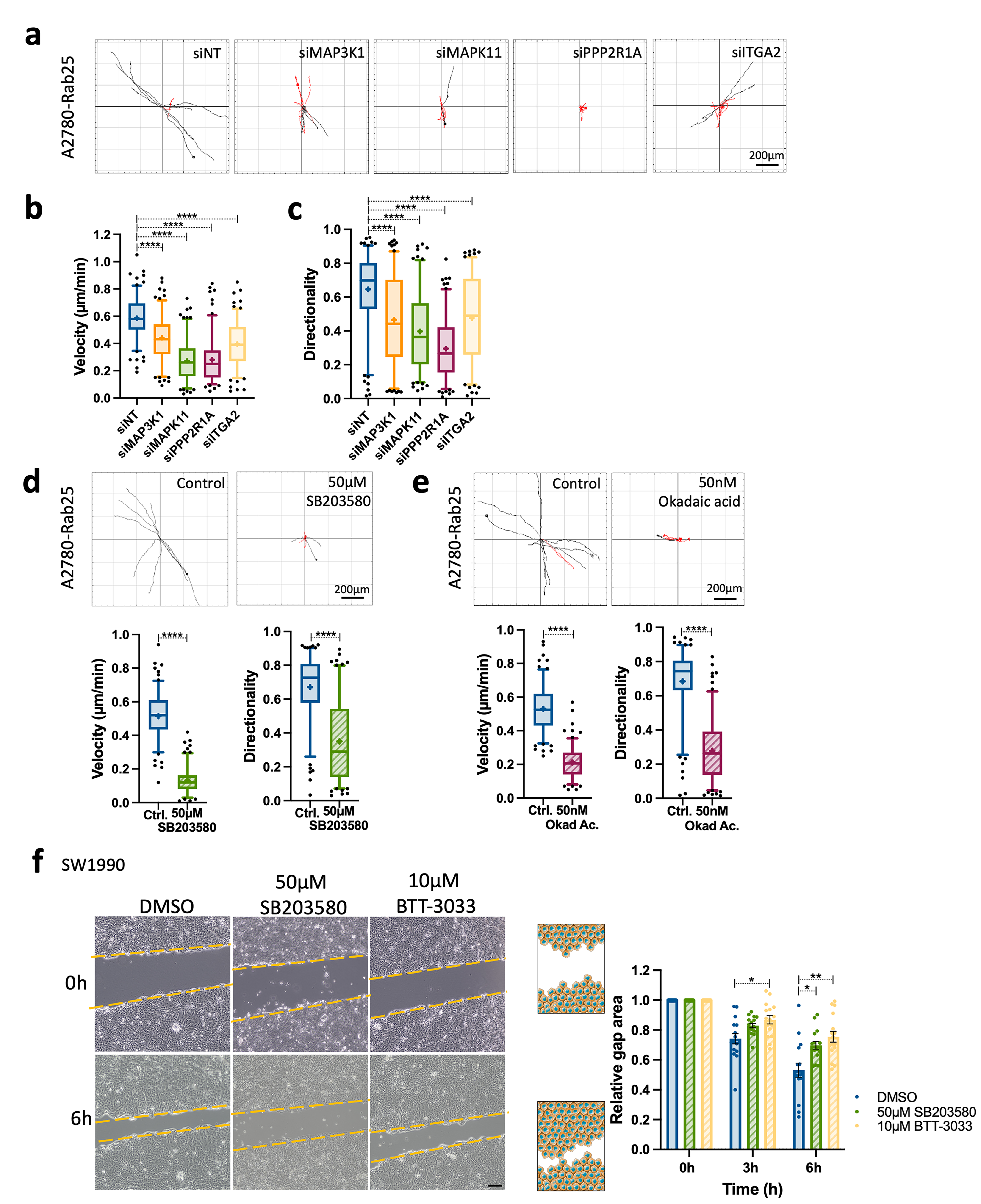

Supplement: S10 Fig — (a–c) A2780-Rab25 cells were transfected with an siRNA targeting MAP3K1 (siMAP3K1), an siRNA targeting MAPK11 (siMAPK11), an siRNA targeting PPP2R1A (siPPP2R1A), an siRNA targeting α2 integrin (si-ITGA2), or a non-targeting siRNA control (siNT), seeded on CDM for 6 h and imaged live with a 10× Nikon Inverted Ti eclipse with Oko-lab environmental control chamber for 17 h. Spider plots show the migration paths of manually tracked cells (directionality >0.5 in black, <0.5 in red). Box and whisker plots represent 5–95 percentile, + represents the mean, dots are <5% and >95%; N = 3 independent experiments. ****p < 0.0001; Kruskal–Wallis test. (d) A2780-Rab25 cells were seeded on CDM for 6 h in the presence of DMSO (Ctrl.) or 50 μM SB203580 and imaged live with a 10× Nikon Inverted Ti eclipse with Oko-lab environmental control chamber for 17 h. Spider plots show the migration paths of manually tracked cells (directionality >0.5 in black, <0.5 in red). Box and whisker plots represent 5–95 percentile, + represents the mean, dots are <5% and >95%; N = 3 independent experiments. ****p < 0.0001; Kruskal–Wallis test. (e) A2780-Rab25 cells were seeded on CDM for 6 h in the presence of the vehicle (water, Ctrl.) and 50 nM Okadaic acid (Okad Ac.) and imaged live with a 10× Nikon Inverted Ti eclipse with Oko-lab environmental control chamber for 17 h. Spider plots show the migration paths of manually tracked cells (directionality >0.5 in black, <0.5 in red). Box and whisker plots represent 5–95 percentile, + represents the mean, dots are <5% and >95%; N = 3 independent experiments. ****p < 0.0001; Kruskal–Wallis test. (f) SW1990 cell confluent monolayers were scratched and overlaid with 0.5 mg/ml collagen I and cells were imaged at 0 h, 3 h, and 6 h. Yellow lines indicate the wound edges. The bar graph shows the normalised relative gap area ± SEM. N = 4 independent experiments. *p ≤ 0.0213, **p = 0.0025; 2-way ANOVA. All the raw data associated with this figure are available in S17 [file pbio.3002930.s010.tif]

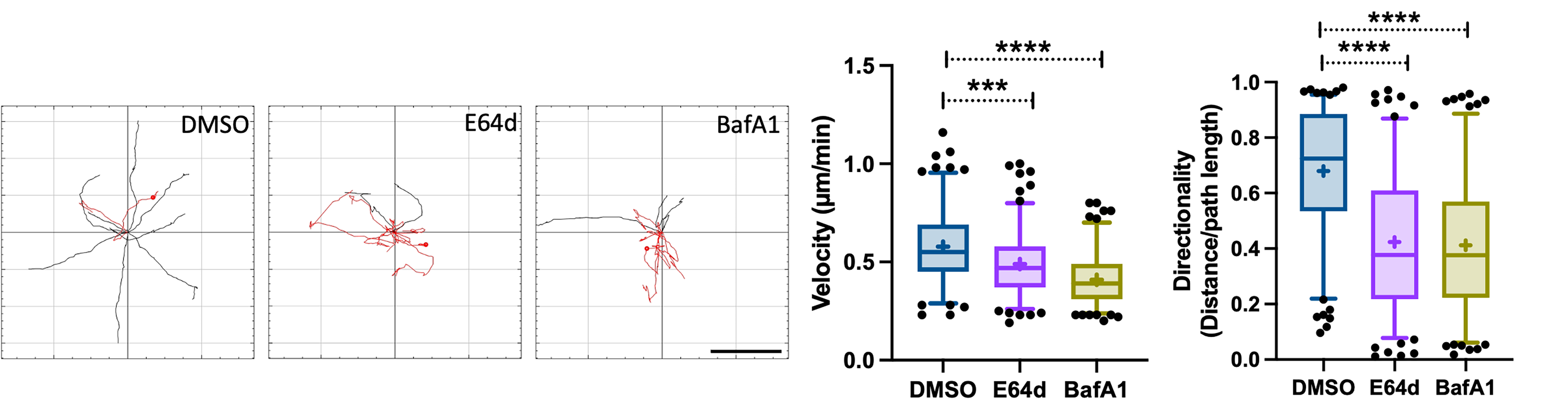

Supplement: S11 Fig — MDA-MB-231 cells were seeded on CDM in the presence of 200 nM Bafilomycin A1 (BafA1), 20 μM E64d, or DMSO control for 6 h and imaged live with a 10× Nikon Inverted Ti eclipse with Oko-lab environmental control chamber for 17 h. Spider plots show the migration paths of manually tracked cells (directionality >0.5 in black, <0.5 in red). Bar, 200 mm. Box and whisker plots represent 5–95 percentile, + represents the mean, dots are <5% and >95%; N = 1 independent experiments. ***p = 0.0002, ****p < 0.0001; Kruskal–Wallis test. All the raw data associated with this figure are available in S18 Data. (TIF) [file pbio.3002930.s011.tif]

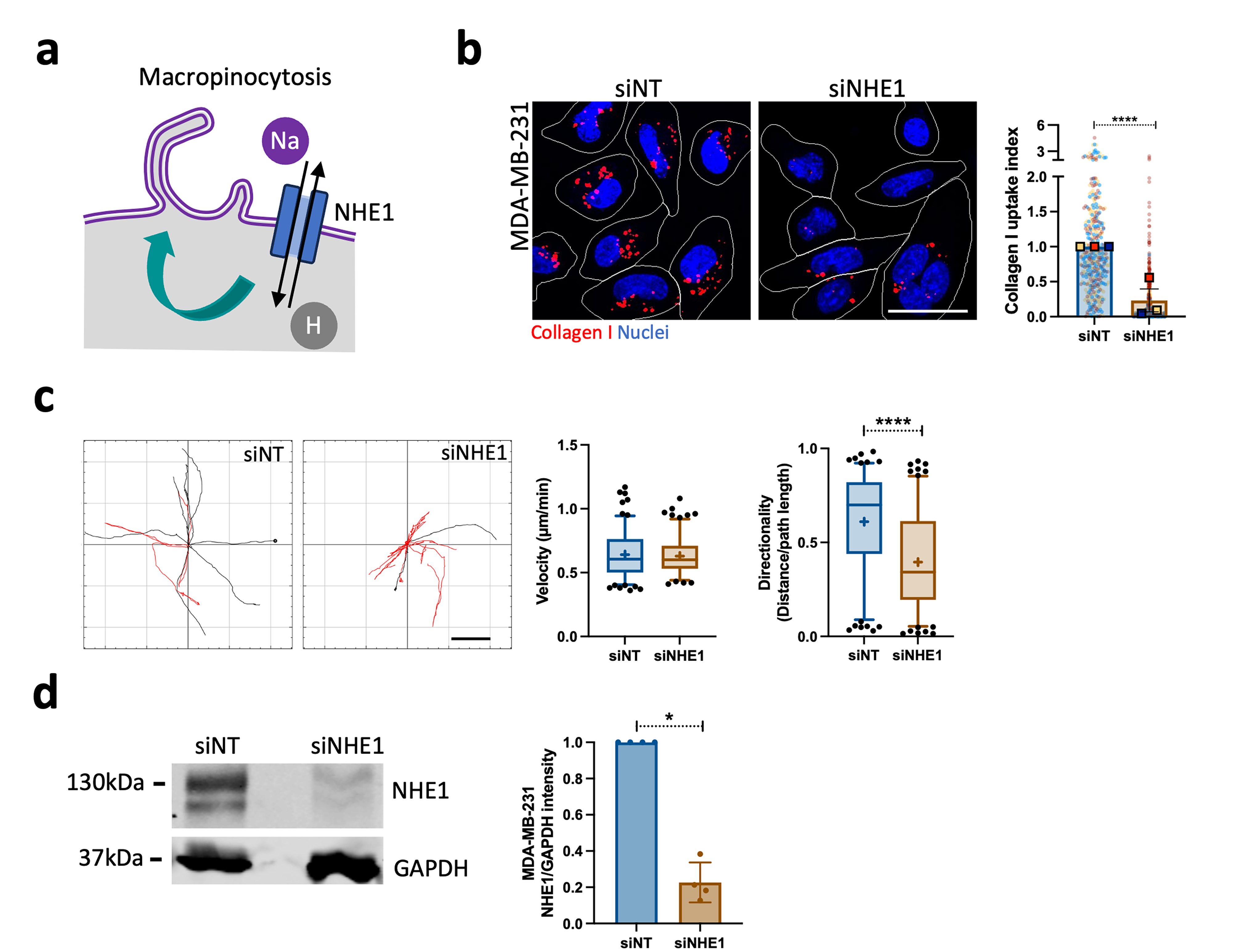

Supplement: S12 Fig — (a) Schematic, NHE1 promotes macropinocytosis. (b) MDA-MB-231 cells were transfected with an siRNA targeting NHE1 (siNHE1) or a non-targeting siRNA control (siNT), seeded on pHrodo-labelled 1 mg/ml collagen I for 6 h, stained with 1 μg/ml Hoechst and imaged live. Scale bar, 30 μm. Collagen I uptake index was calculated with Image J. Values represented are normalised mean + SD from N = 3 independent experiments; ****p < 0.0001; Mann–Whitney test. (c) MDA-MB-231 cells were transfected as in (a), seeded on CDM and imaged live with a 10× Nikon Inverted Ti eclipse with Oko-lab environmental control chamber for 17 h. Spider plots show the migration paths of manually tracked cells (directionality >0.5 in black, <0.5 in red). Bar, 200 mm. Box and whisker plots represent 5–95 percentile, + represents the mean, dots are <5% and >95%; N = 3 independent experiments. ****p < 0.0001; Kruskal–Wallis test. (d) Cells were transfected as in (a) for 72 h, lysed and NHE1 and GAPDH protein levels were measured by western blotting. Data are presented as the normalised mean ± SD; N = 4 independent replicates. *p = 0.0286; Mann–Whitney test. All the raw data associated with this figure are available in S19 Data. (TIF) [file pbio.3002930.s012.tif]

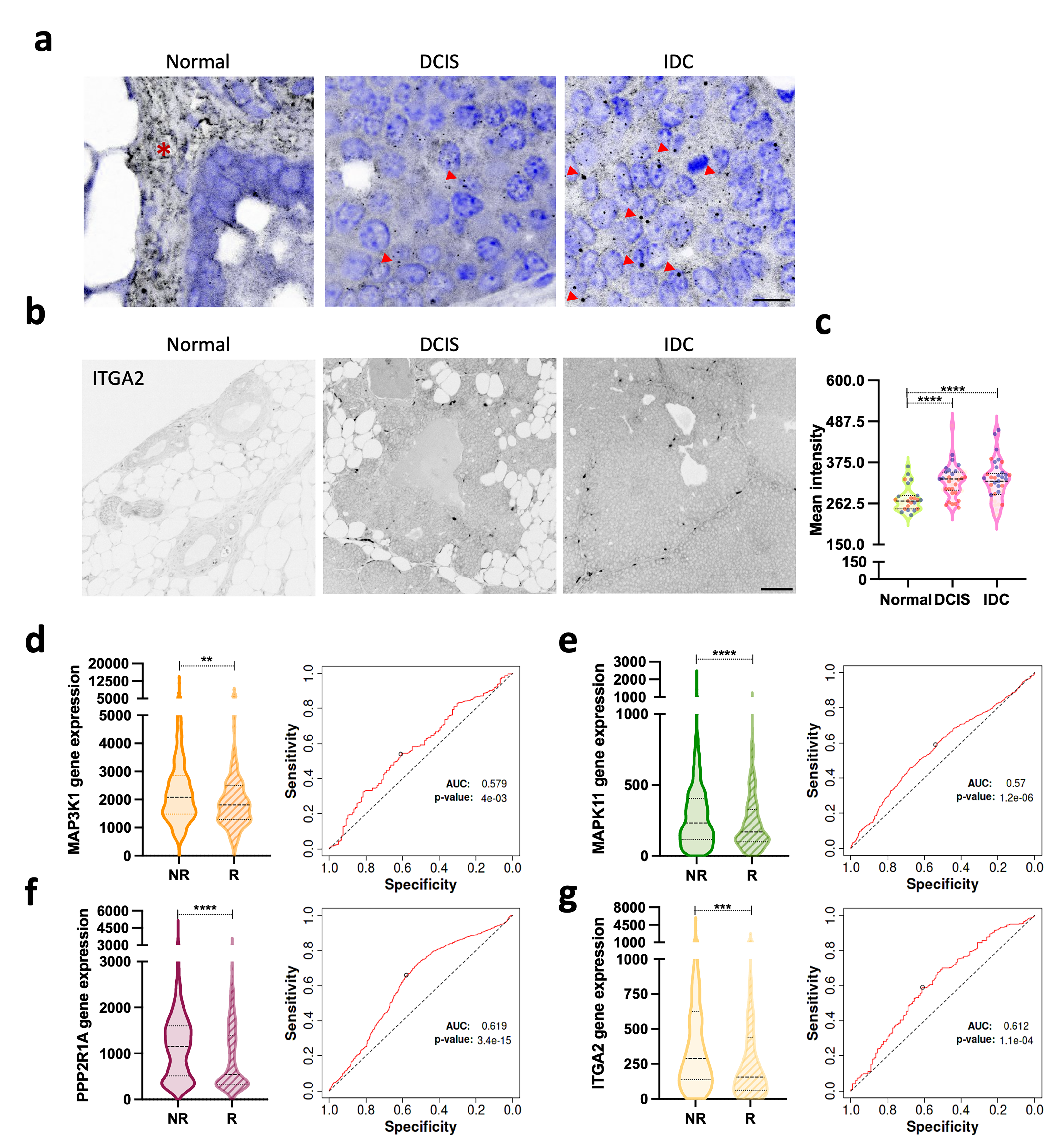

Supplement: S13 Fig — (a, b) Tissue sections from polyoma middle T-derived mouse mammary tumours were stained for collagen I (black) and nuclei (blue) (a) or α2 integrin (ITGA2, b); * highlights the stroma, arrow-head indicate collagen I-positive vesicles. Scale bar, 10 μm (a) and 50 μm (b). (c) α2 integrin mean intensity was quantified with Image J; N = 3 independent experiments. ****p < 0.0001; Kruskal–Wallis test. (d–g) RNA sequencing data and ROC analysis for MAP3K1 (a), MAPK11 (b), PPP2R1A (c), and α2 integrin (ITGA2, d) from chemoresistant (non-responder (NR)) and chemosensitive (responder (R)) breast cancer tumours. **p = 0.0090; ***p = 0.0002; ****p < 0.0001; Mann–Whitney test. All the raw data associated with this figure are available in S20 Data. (TIF) [file pbio.3002930.s013.tif]
