# Supplementary material for: Novel kinase regulators of extracellular matrix internalisation identified by high-content screening modulate invasive carcinoma cell migration
Source: PLoS Biol. 2024 Dec 12;22(12):e3002930. doi: 10.1371/journal.pbio.3002930 (PMC11637276; doi:10.1371/journal.pbio.3002930)

Fig.3f

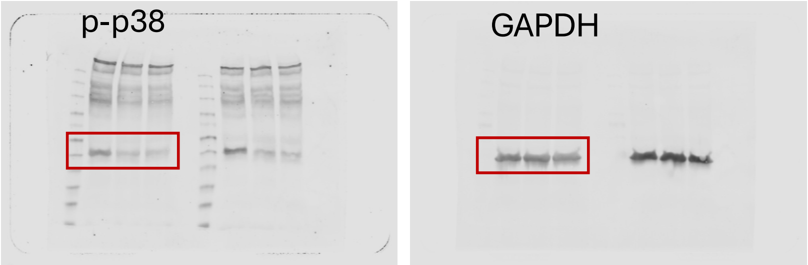

Extended Data Fig.2g

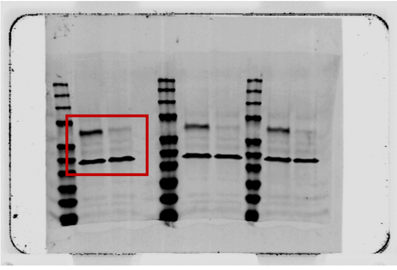

Extended Data Fig.4g

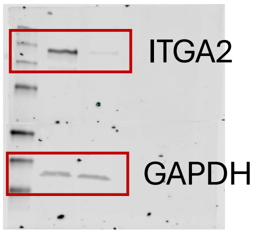

Extended Data Fig.6j,k

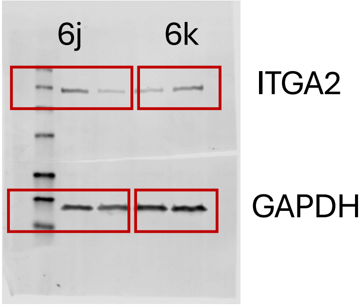

Extended Data Fig.8e

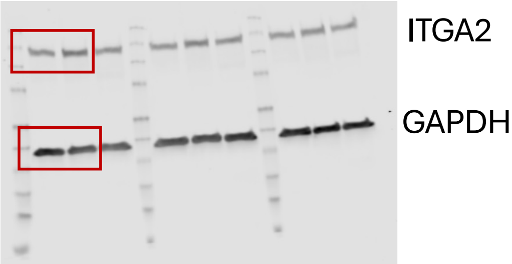

Extended Data Fig.12d

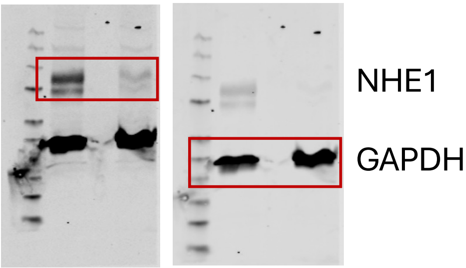

Supplement: S1 Raw Images — (PDF) [file pbio.3002930.s022.pdf]
