# Supplementary material for: Novel kinase regulators of extracellular matrix internalisation identified by high-content screening modulate invasive carcinoma cell migration
Source: PLoS Biol. 2024 Dec 12;22(12):e3002930. doi: 10.1371/journal.pbio.3002930 (PMC11637276; doi:10.1371/journal.pbio.3002930)
Supplement: Attachment — Submitted filename: Response_to_reviewers_Llanses_Martinez.pdf [file pbio.3002930.s045.pdf]

We would like to thank all the reviewers for their positive evaluations of our work and constructive comments. We have now addressed all the concerns raised and modified the manuscript accordingly (all the changes are highlighted in blue in the manuscript file).

***Reviewer #1***

**The data look interesting but what seems to be lacking are controls for the silencing efficacy of the top targets in the screen**.

We used integrin β1 and PAK1 as positive controls in the screen. We have now included the integrin β1 staining in the screening plate, to confirm the knockdown efficiency (**extended data fig. 2f**). In addition, Western Blotting experiments confirmed a >75% reduction in PAK1 levels upon siRNA transfection (**extended data fig. 2g**).

**It would be good to show the results for the 2 independent MAPK11 siRNAs**.

We have now used 3 individual siRNAs targeting MAPK11 and we showed that they all reduced collagen I uptake (**extended data fig. 7a,b**). We confirmed the knock-down efficiency using qPCR (**extended data fig. 7c**).

**Would be important to show p38-inhibition (phospho-wb) for the experiments where inhibitors are used**.

We have now included a western blot analysis showing significant reduction in p38 phosphorylation in cells treated with SB203580 and SB202190 (**figure 3f**).

**Use more than 1 siRNA for PP2A**.

We have now included a heatmap showing the effect of the knockdown of the different PP2A subunits on ECM uptake (**extended data fig. 3a,b**), demonstrating that PPP2R1A has the strongest effect on ECM uptake. PPP2R1A is a core PP2A subunit, and its loss has been shown to destabilise the whole PP2A complex (Kauko et al., 2020). In the deconvolution experiment (**figure 1f**), we are showing for individual siRNA sequences targeting PPP2R1A.

**Is the ability to take up ECM dependent on ECM proteolytic degradation**?

In our recent publication (Nazemi et al., 2024), we assessed the role of matrix metalloproteases (MMP) in ECM uptake and ECM-dependent cell proliferation under starvation by treating MDA-MB-231 cells with the broad spectrum MMP inhibitor GM6001. We found that MMP inhibition did not prevent ECM uptake nor ECM-dependent cell growth, consistent with previous findings in the literature (Yamazaki et al., 2020). We are currently characterising the role of secreted cathepsins in controlling ECM uptake as a separate project in our lab, and preliminary data suggest that they might be involved. We feel this point is outside the scope of the current manuscript.

**Okadaic acid treatment has the tendency to detach cells from the ECM. Was this observed here/controlled for**?

We agree with the reviewer that this effect could indeed affect the interpretation of our results. We'd like to point out that in this study, we used relatively low concentrations (50nM) compared to some published work (up to 300nM). To assess the effect of okadaic acid on cell morphology, we measured the aspect ratio of MDA-MB-231 and A2780-Rab25 cells migrating on CDM and found that okadaic acid treatment and PPP2R1A downregulation resulted in a similar reduction in aspect ratio, representative of more rounded cells (**extended data fig. 3d-g**), but we did not detect cell-ECM detachment. To note, the effect on cell morphology was more profound in the cell migration experiments, where the cells are sparser, compared to the ECM uptake experiments, where the cells are more confluent.

**Is the ECM endocytosis driven migration linked to the ability of the cells to degrade the endocytosed material in their lysosomes (to provide nutrients for the cell)? Or is it more a mechanism of ECM remodelling to enable invasion?**
This is a very important and interesting point. To address this, we used lysosomal inhibitors (E64d, a broad spectrum cathepsin inhibitor) and bafilomycin A1 (a vATPase inhibitor, which prevent lysosomal degradation by blocking acidification). We confirmed that the treatment with both inhibitors resulted in an accumulation of intracellular collagen I (**extended data fig. 8a**), indicative of inhibition of lysosomal degradation. Interestingly, both inhibitors also significantly reduced velocity and directionality of cell migration on cell-derived matrices (**extended data fig. 11**), suggesting that ECM degradation is required for migration through complex matrices.

**Finally, what is the faith of the integrin vs. the ECM ligand? Are both degraded or is the integrin recycled?**
We have included new data showing that both ECM ligand (collagen I) and $\alpha2\beta1$ integrins are degraded in the lysosomes, as lysosomal inhibition resulted in an increase in the internal pool, as well as total $\alpha2\beta1$ integrin levels (**extended data fig. 8**).

***Reviewer #2***
**<u>Major comments</u>:**
**Mechanistic insight into how these kinases and this specific regulatory subunit of the PP2 phosphatase is involved in this process. What are the targets of these kinases and phosphatase? Do they regulate a2b1-integrin phosphorylation or trafficking?**
We don't believe that $\alpha2\beta1$ is a target of p38, as we did not find any evidence of this in previous p38 phospho-proteomic studies, while $\alpha2\beta1$ has been reported as an upstream regulator of p38. We agree with the reviewer that including more details on the potential p38 targets modulating ECM uptake and migration would be beneficial. We also agree with the reviewer that performing the extensive phospho-proteomic approach and target validation will constitute an entirely different project and this point should not preclude the publication of this paper. The sodium/proton channel NHE1 has been reported as a p38 target (Khadler et al., 2001; Grenier et al., 2008), and it is also a well-known regulator of macropinocytosis. We have now included new data showing that NHE1 is required for both ECM uptake and breast cancer cell migration on fibroblast-generated cell-derived matrices (**extended data fig. 12**).

**It is quite an overstatement to conclude from a 1-to-1 comparison between NMuMG cells and one cell line derivative of PyMT tumour that "these data indicate that ECM internalisation and degradation is upregulated in invasive breast cancer." Either soften this statement (e.g. 'ECM internalisation was higher in PyMT cancer cells than NMuMG normal breast cells'), or provide experimental evaluation across a range of normal versus cancer cells in vitro and using in vivo systems.**
We soften the statement, and we described in more details the evidence that we collected from the MCF10 series of cell lines (non-transformed, non-invasive and metastatic cell lines) in the results and discussion.

**It is not clear that the authors are comparing like for like. In extended Data Figure 1A, B, In NMuMG cells, these are islands of cells with tight cell-cell compaction, whereas PyMT#1 appear as less adherent and compact cells with discontinuous cell-cell adhesions. While it is still appropriate to compare uptake normalised by area of cells, can the authors provide examination of what the ECM update is upon**

**similar cell states, i.e. when both cell types are colonies versus elongated single or chains of cells? This would delineate whether differences are due to cell-cell contact or not, or bona fide differences in ECM uptake despite such different morphologies.**
Similar changes in ECM uptake were observed in the MCF10 series of cell lines (Nazemi et al., 2024), where there is no clear morphological difference between the cell lines, indicating that cell-cell adhesion or elongation do not play a significant role here. We have included a statement about this in the discussion.

**Throughout, the authors use cartoons of 3D culture of NMuMG, PyMT1 cells, breast to indicate MDA-MB-231 cells, a picture of a mouse, and a pancreas in attempt to orient the reader. This is very confusing as, for example in Extended Data Fig. 1A, B, these suggest 3-Dimensional spheroid cultures, when these are actually isolated cells or, when what is being demonstrated are not 3-Dimensional, but rather are 2D cells inside ECM.**
We apologise for creating confusion with the cartoons, we have now removed all the small diagrams, including cartoons representing normal, DCIS and invasive cells, as well as cartoons representing breast, ovarian, pancreatic and mouse cells. Diagrams have been replaced by adding the name of the cell line, where multiple cell lines are present in the same figure.

**Why did the authors perform the screen only two times (not trying to diminish the effort here!), when thrice may have helped with statistical analyses? The authors provide significance values for Reactome pathway assessment. How appropriate it is for the presentation of these from only two independent replicates?**
We have now clarified how hits were selected in the methods section, accompanied by references of impactful publication screenings where biological duplicates have been previously used, including Sharma and Rao, Nat Immunol 2009 and Chia et al., Nature 2010.

**How have the authors assessed whether, and if so to what extent, their cell segmentation is accurate? Can the authors provide evidence for this? For instance, in Figure 2b, this appears to be error-prone, at least for MDA-MB-231 cells.**
We apologise for the confusion caused; we have now clarified how cells are detected in the methods section: Cells were imaged using a 60x Nikon A1 confocal microscope. For these experiments, cells were stained for a membrane protein, which is not shown in the images for better visualisation of the uptake. For live imaging uptake, the outline of the cells was visible, therefore being used to calculate the cell area. Confocal experiments were analysed manually.

**Can the authors show in vivo that they can see internalised ECM, such as in sections of breast cancer models, internal pools of ECM in the invasive front of tumours?**
We have included new data showing that collagen I positive vesicles can be visualised in sections of breast cancer models, while they are completely absent in the normal mammary gland (**extended data fig. 13a**).

**<u>Minor comments</u>:**

**The colour schemes that the authors use throughout are not colourblind friendly, and somewhat difficult to follow even for colour-able readers.**
We apologise that the colours chosen in the plots could be difficult to distinguish for colourblind people. We have now changed the colour from all the super-plot graphs in the manuscript, so they are colourblind friendly, we have tested this by using an online website simulator (https://pilestone.com/pages/color-blindness-simulator-1#), which shows how graphs are visualised by the diverse spectrum of colourblind readers.

**Extended Data Fig. 3 g,h (ITGA2+ITGB1 KD validation) are not mentioned in the main text.**
Thank you for pointing this out. We have now included previous Extended Data Fig. 3g (now **extended data fig. 4g**) in the result section. Extended data Fig 3h (β1 integrin knockdown) was mentioned together with Extended data Fig 3a (β1 integrin knockdown on matrigel uptake) to facilitate the reading in section 'ECM internalisation is dependent on α2β1 integrin'.
