# Supplementary material for: Novel kinase regulators of extracellular matrix internalisation identified by high-content screening modulate invasive carcinoma cell migration
Source: PLoS Biol. 2024 Dec 12;22(12):e3002930. doi: 10.1371/journal.pbio.3002930 (PMC11637276; doi:10.1371/journal.pbio.3002930)
Supplement: Attachment — Submitted filename: 143918_1_rebuttal_3004408_sct12l.docx [file pbio.3002930.s043.docx]

**Manuscript number:** RC-2024-02371

**Corresponding author(s):** Elena, Rainero

## 1. General Statements

We would like to thank both reviewers, for highlighting that our work is a ‘careful mechanistic and functional investigation’ and that the data are ‘clear, convincing and appropriately analysed’. We appreciated that our work was recognised to be important the ‘cell signalling, ECM, and migration field’ and ‘may be translationally relevant’. Below we list how we have addressed or are planning to address all the concerns raised by the reviewers. All the changes are marked in blue in the text.

## 2. Description of the planned revisions

*MAPK11 data in figure 1f (deconvolution)*.

We agree with the reviewer that this is an important point. MAPK11 was not initially included in the deconvolution list, as it was a weak hit from the screen. We have now used the 4 individual siRNAs which are the components of the smart pool used in the screen, and we measured collagen I internalisation in MDA-MB-231 breast cancer cells. Preliminary data indicate a statistically significant reduction in collagen I uptake in 3 out of 4 sequences tested (revision figure 1). The efficiency of the siRNAs in reducing MAPK11 levels will be measured by qPCR.

*Show p38 inhibition (WB) for the experiments in which the inhibitors were used*.

To assess the efficacy of SB203580 at inhibiting p38 signalling, we will assess the phosphorylation of the p38 target ATF2, as previously described (Ivaska et al., 1999).

*Is ECM endocytosis-driven migration linked to the ability of the cells to degrade the endocytosed material in their lysosomes? Or is it more a mechanism of ECM remodelling to enable invasion*? [Reviewer 1]. Not clear whether ECM uptake actually fuels/is required for invasion, or whether it is simply a consequence [Reviewer 2].

We thank the reviewers for raising this important point. Indeed, it is possible that ECM uptake impacts on both these processes. To elucidate this, we will treat the cells with Bafilomycin A1, to prevent lysosomal acidification and degradation and assess the migratory ability of MDA-MB-231 cells. If ECM endocytosis-driven migration is an ECM-remodelling mechanism, we expect cell migration not to be affected by the presence of Bafilomycin A1; on the contrary, if ECM lysosomal degradation is required, we expect Bafilomycin A1 treatment to impair cell migration.

*What is the faith of the integrin vs ECM ligand*?

While we showed that internalised ECM components are degraded in the lysosomes, we do not know the faith of the integrin receptor. To measure integrin α2β1 degradation, we will monitor its levels by Western Blotting in the presence of cycloheximide on both plastic and 1mg/ml collagen I, which drives α2β1 internalisation. In addition, we will measure α2β1 internal pool in the presence of E64d, which we showed prevented the degradation of internalised collagen I.

*Mechanistic insight into how these kinases and this specific regulatory subunit of the PP2 phosphatase is involved in this process. What are the targets of these kinases and phosphatase? Do they regulate a2b1-integrin phosphorylation or trafficking*?

We don’t believe that a2b1 is a target of p38, as we did not find any evidence of this in p38 phosphoproteomic studies, while a2b1 has been reported as an upstream regulator of p38. We agree with the reviewer that including more details on the potential p38 targets modulating ECM uptake and migration would be beneficial. We also agree with the reviewer that performing the extensive phospho-proteomic approach and target validation will constitute an entirely different project and this point should not preclude the publication of this paper. The sodium/proton channel NHE1 has been reported as a p38 target (Khadler et al., 2001; Grenier et al., 2008), and it is also a well-known regulator of macropinocytosis. Therefore, here we will investigate whether NHE1 is also phosphorylated by p38 in our system and whether it is required for ECM uptake and cell migration. We have already established that treatment with the NHE1 inhibitor EIPA significantly reduced ECM uptake in MDA-MB-231 cells (Nazemi et al., 2024). PP2A has been shown to dephosphorylate p38, therefore we will confirm this in our system by measuring p38 levels by western blotting.

## 3. Description of the revisions that have already been incorporated in the transferred manuscript

*Controls for the silencing efficiency in the screen are missing*.

We used integrin β1 and PAK1 as positive controls in the screen. We have now included the integrin β1 staining in the screening plate, to confirm the knock down efficiency (extended figure 2f). In addition, Western Blotting experiments confirmed a >75% reduction in PAK1 levels upon siRNA transfection (extended figure 2g).

*Show p38 inhibition (WB) for the experiments in which the inhibitors were used*.

Phospho-p38 WB has been extensively used to assess the efficiency of SB202190 treatment, therefore, we performed similar experiments in MDA-MB-231 and found that treatment with SB202190 almost completely abolished p38 phosphorylation induced collagen I adhesion (figure 3f).

*Use more than 1 siRNA for PP2A*.

We are now including a heatmap showing the effect of the knock down of the different PP2A subunits on ECM uptake (extended figure 3a,b), demonstrating that PPP2R1A has the strongest effect on ECM uptake. PPP2R1A is a core PP2A subunit and its loss has been shown to destabilise the whole PP2A complex (Kauko et al., 2020). In the deconvolution experiment (figure 1f), we are showing for individual siRNA sequences targeting PPP2R1A.

*Okadaic acid has the tendency to detach cells from the ECM*.

We agree with the reviewer that this effect could indeed affect the interpretation of our results. We’d like to point out that in this study, we used relatively low concentrations (50nM) compared to some published work (up to 300nM). To assess the effect of okadaic acid on cell morphology, we measure the aspect ratio of MDA-MB-231 and A2780-Rab25 cells migrating on CDM and found that okadaic acid treatment and PPP2R1A downregulation resulted in a similar reduction in aspect ratio, representative of more rounded cells (extended figure 3d-ga,b), but we did not detect cell-ECM detachment. To note, the effect on cell morphology was more profound in the cell migration experiments, where the cells are sparser, compared to the ECM uptake experiments, where the cells are more confluent.

*It is quite an overstatement to conclude from a 1-to-1 comparison between NMuMG cells and one cell line derivative of PyMT tumour that "these data indicate that ECM internalisation and degradation is upregulated in invasive breast cancer." Either soften this statement (e.g. 'ECM internalisation was higher in PyMT cancer cells than NMuMG normal breast cells'), or provide experimental evaluation across a range of normal versus cancer cells in vitro and using in vivo systems.*

We soften the statement, and we described in more details the evidence that we collected from the MCF10 series of cell lines (non-transformed, non-invasive and metastatic cell lines) in the results and discussion.

*It is not clear that the authors are comparing like for like. In extended Data Figure 1A, B, In NMuMG cells, these are islands of cells with tight cell-cell compaction, whereas PyMT1 appear as less adherent and compact cells with discontinuous cell-cell adhesions. While it is still appropriate to compare uptake normalised by area of cells, can the authors provide examination of what the ECM update is upon similar cell states, i.e. when both cell types are colonies versus elongated single or chains of cells? This would delineate whether differences are due to cell-cell contact or not, or bona fide differences in ECM uptake despite such different morphologies.*

Similar changes in ECM uptake were observed in the MCF10 series of cell lines, where there is no clear morphological difference between the cell lines, indicating that cell-cell adhesion or elongation do not play a significant role here. We have included a statement about this in the discussion.

*Throughout, the authors use cartoons of 3D culture of NMuMG, PyMT1 cells, breast to indicate MDA-MB-231 cells, a picture of a mouse, and a pancreas in attempt to orient the reader. This is very confusing as, for example in Extended Data Fig. 1A, B, these suggest 3-Dimensional spheroid cultures, when these are actually isolated cells or, when what is being demonstrated are not 3-Dimensional, but rather are 2D cells inside ECM.*

We apologise for creating confusion with the cartoons, we have now removed all the small diagrams, including cartoons representing normal, DCIS and invasive cells, as well as cartoons representing breast, ovarian, pancreatic and mouse cells. Diagrams have been replaced by adding the name of the cell line, where multiple cell lines are present in the same figure.

*Why did the authors perform the screen only two times (not trying to diminish the effort here!), when thrice may have helped with statistical analyses? The authors provide significance values for Reactome pathway assessment. How appropriate it is for the presentation of these from only two independent replicates?*

We have now clarified how hits were selected in the methods section, accompanied by references of impactful publication screenings where biological duplicates have been previously used, including Sharma and Rao, Nat Immunol 2009 and Chia et al., Nature 2010.

*How have the authors assessed whether, and if so to what extent, their cell segmentation is accurate? Can the authors provide evidence for this? For instance, in Figure 2b, this appears to be error-prone, at least for MDA-MB-231 cells.*

We apologise for the confusion caused, we have now clarified how cells are detected in the methods section: Cells were imaged using a 60x Nikon A1 confocal microscope. For these experiments, cells were stained for a membrane protein, which is not shown in the images for better visualisation of the uptake. For live imaging uptake, the outline of the cells was visible, therefore being used to calculate the cell area. Confocal experiments were analysed manually.

*The colour schemes that the authors use throughout are not colourblind friendly, and somewhat difficult to follow even for colour-able readers.*

We apologise that the colours chosen in the plots could be difficult to distinguish for colorblind people. We have now changed the colour from all the superplot graphs in the manuscript, so they are colourblind friendly, we have tested this by using an online website simulator (https://pilestone.com/pages/color-blindness-simulator-1#), which shows how graphs are visualised by the diverse spectrum of colorblind readers.

Extended Data Fig. 3 g,h (ITGA2+ITGB1 KD validation) are not mentioned in the main text.

Thank you for pointing this out. We have now included previous Extended Data Fig. 3g (currently 4g) in the result section. Extended data Fig 3h (β1 integrin knockdown) was mentioned together with Extended data Fig 3a (β1 integrin knockdown on matrigel uptake) to facilitate the reading in section ‘ECM internalisation is dependent on α2β1 integrin’.

## 4. Description of analyses that authors prefer not to carry out

*Is the ability to take up ECM dependent on ECM proteolytic degradation*?

In our recent publication (Nazemi et al., 2024), we assessed the role of matrix metalloproteases (MMP) in ECM uptake and ECM-dependent cell proliferation by treating MDA-MB-231 cells with the broad spectrum MMP inhibitor GM6001. We found that MMP inhibition did not prevent ECM uptake nor ECM-dependent cell growth, consistent with previous findings in the literature (Yamazaki et al., 2020). We are currently characterising the role of secreted cathepsins in controlling ECM uptake as a separate project in our lab, and preliminary data suggest that they might be involved. We feel this point is outside the scope of the current manuscript.
